# Reconstructing the modular recombination history of Staphylococcus aureus phages

**DOI:** 10.1186/1471-2105-14-S15-S17

**Published:** 2013-10-15

**Authors:** Krister M Swenson, Paul Guertin, Hugo Deschênes, Anne Bergeron

**Affiliations:** 1 LaCIM, Université du Québec à Montréal, Canada; 2 Département de mathématiques, Collège André Grasset, Montréal, Canada

## Abstract

**Background:**

Viruses that infect bacteria, called *phages*, are well-known for their extreme mosaicism, in which an individual genome shares many different parts with many others. The mechanisms for creating these mosaics are largely unknown but are believed to be recombinations, either illegitimate, or partly homologous. In order to reconstruct the history of these recombinations, we need to identify the positions where recombinations may have occurred, and develop algorithms to generate and explore the possible reconstructions.

**Results:**

We first show that, provided that their gene order is co-linear, genomes of phages can be aligned, even if large parts of their sequences lack any detectable similarity and are annotated *hypothetical proteins*. We give such an alignment for 31 *Staphylococcus aureus *phage genomes, and algorithms that can be used in any similar context. These alignments provide the datasets needed for a combinatorial study of recombinations. We next reconstruct the most likely recombination history of the set of 31 phages, under the hypothesis that recombinations are partly homologous. This history relies on the computational identification of missing phages.

**Conclusions:**

This first combinatorial study of modular recombinations acts as a proof of concept. We show that alignments of whole genomes are feasible for large sets of phages, and that this representation yields data that can be used to reconstruct parts of the evolutionary history of these organisms.

## Background

Chromosome recombinations within species are a powerful way to create variation, eventually leading to selection. In some species, such as humans, recombinations exchange highly similar chromosome segments differing at isolated positions called Single Nucleotide Polymorphisms. The identification and ordering of these events gave rise to the multiple population genetics models that try to reconstruct the history of the species [1-3].

In a very different world, phages often use recombination as a radical invention process: "Let's exchange our legs for blades, and see if we fare better." Legs and blades have no detectable similarity but have the same function, in this case *running*. We will call these events *modular recombinations *in order to distinguish them from (similar) chromosomal recombinations.

**Figure 1:**
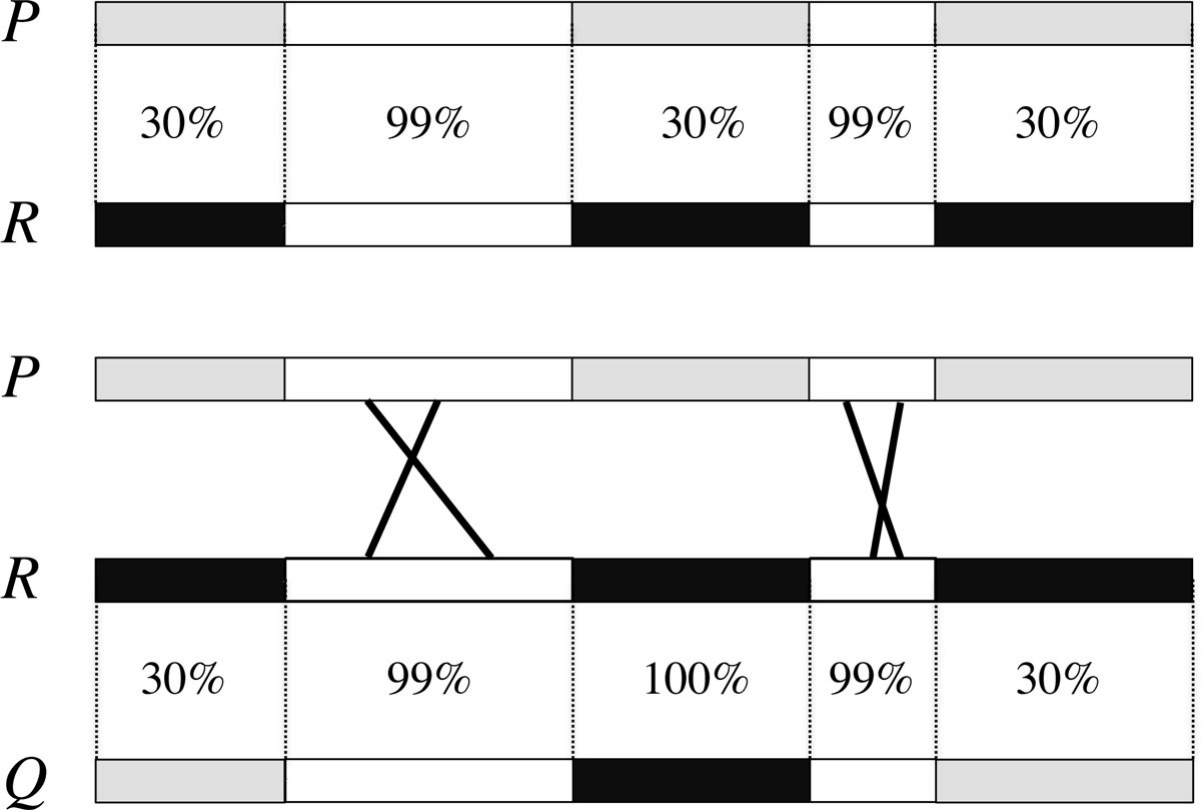
**Phage recombination**. Top: comparison of phages *P *and *R *along their sequence reveals an alternating pattern of highly similar and divergent regions. Bottom: a modular recombination event between phages *P *and *R*. It uses two pairs of highly similar regions of phages *P *and *R *to produce phage *Q*.

The mechanisms allowing modular recombination are still debated. Some argue that new phage architectures are selected from a large number of random breaks and repairs that mix different genomes, a process called *illegitimate recombination *[4]. Others, such as [5], suggest that divergent sequences are transferred from one phage to the other using flanking homologous sequences, as illustrated in Figure 1. The authors give compelling evidence of the occurrence of this type of recombination in the family of phages that infect *Staphylococcus aureus *and in various other families.

Our goal is the detection, reconstruction and ordering of modular recombination events, using the model proposed in [5]. We apply our methods to medium length *siphoviruses *(around 40,000 bp) that are by far the most common in databases, and the most studied since they seem to be partly responsible for virulence factors associated with antibiotic resistant bacteria [6].

### Phage structure and modular theory

When the whole sequences of two phages involved in recombination events are compared, there is an alternating pattern of similar and divergent sequences. This phenomenon was observed in the last century and gave rise to the *modular theory *[7] which postulates that phages are assemblages of modules that carry identical biological function with possibly different tools. An example of such a module is the *integrase *gene, that allows the phage to insert its own genome into the circular genome of its bacterial hosts. The nucleotide sequence of an integrase is highly correlated with the specific bacterial gene where the insertion will occur: in *Staphylococcus aureus*, around 10 different integration sites are known [8], with corresponding integrase genes. Two otherwise identical phages may differ, for example, only in their integrase genes.

We will refer to the different implementations of a module as *variants *of that module. The number of modules in a phage varies according to the various informal definition of module: each phage species has 5 to 10 main modules, but these can be subdivided into a few dozen. The number of known variants typically ranges from 4 to 10. A standard organization of modules for siphoviruses is the following:

1. Lysogeny. This module contains the integrase gene.

2. DNA metabolism.

3. Head morphology and DNA packaging.

4. Tail assembly.

5. Lysis.

A last characteristic of co-linear phages is that multiple copies of their linear genome are physically concatenated during their killing cycle. Recombinations that occur at this stage may involve the end of one of the concatenated copies with the beginning of the next copy: to analyze recombinations occurring at that stage, we need to make the hypothesis that the phage genomes are circular, thus the list of modules given above must be interpreted as circular. The integrase gene, for example, is often the first gene in a siphoviruses genome sequence, but many begin with tail assembly genes. For simplicity, we will assume that each phage sequence is described by a linear sequence that begins with its integrase.

In order to detect possible modular recombinations, it is necessary to know the position and variants of each module in a given set of phages. This knowledge is based on the notion of *alignment *of phages.

### Alignments of phage genomes

Phage genomes are not alignable in the usual bioinformatics sense, because many variants of the same module are not comparable in terms of nucleotide composition and/or length. However, when comparing different phages, we want the different variants of the same module to be 'aligned'. This paradox is solved by the following definition:

**Definition 1**. *An *alignment *of a set of n co-linear phage sequences is the identification of k positions of each phage sequence, at least one of them immediately before the first gene, subdividing each sequence into k possibly empty segments. We refer to the sets of n segments at the same position as a *module *of the alignment, and group the non-empty segments within a module into *variants.

Note that Definition 1 does not contain explicit rules to tell when two segments are the 'same' variant. In practice, very high sequence identity is the standard, since these will be the segments that determine the homologous participants of the modular recombination events.

An alignment uses both sequence similarity and functional annotation of phage genes: for well-annotated genes, the modules of the alignment correspond to biological reality; for unannotated genes -- which are currently the vast majority -- the modules of the alignment are essentially constructed using sequence similarity. These techniques are described in more detail in the Methods section.

Figure 1 shows a toy example of three phages that share five modules. Two of the modules have a single white variant, that appears in highly similar (99%) forms over the three phages. The other three modules have two different variants, a black variant and a gray variant.

### Mathematical model of modular recombinations

A *phage *is represented by a list of integers *a*_1_, *a*_2_, . . . , *a_k_*. Each integer *a_i _*≥ 1 represents a variant of module *i*, and the list is circular, in the sense that module *k *is adjacent to module 1. The number of modules *k *is fixed. The number of variants of each module is a constant, though the number of variants may differ from one module to the next. When a module is absent in a phage, its variant number is set to 0, thus our model of modular recombination can represent deletions.

For example, consider the following four phages with 6 modules:

$$\begin{array}{ccccccc} A= & 2 & 1 & 4 & 3 & 2 & 1\\ B= & 2 & 2 & 4 & 2 & 0 & 2\\ C= & 2 & 2 & 4 & 3 & 2 & 1\\ D= & 2 & 2 & 4 & 2 & 2 & 1 \end{array}$$

A *modular recombination *between two phages:

$$\begin{array}{c} a_{1}\ldots a_{i}\ldots a_{j}\ldots a_{k}\;\text{and}\\ b_{1}\ldots b_{i}\ldots b_{j}\ldots b_{k} \end{array}$$

exchanges the segments *a_i _*. . . *a_j _*and *b_i _*. . . *b_j_*, provided that *a_i _= b_i_, a_j _= b_j_*, and **both are non-zero**. The modules corresponding to *a_i _*and *a_j _*are called the *flanking modules *of the recombination. For example, a recombination between phages *A *and *B*, with *i *= 1 and *j *= 3, may produce phage *C*:

$$\begin{array}{l} \underline{\begin{array}{lllllll} A= & 2 & 1 & 4 & 3 & 2 & 1\\ B= & 2 & 2 & 4 & 2 & 0 & 2 \end{array}}\\ \begin{array}{lllllll} C= & 2 & 2 & 4 & 3 & 2 & 1 \end{array} \end{array}$$

Alternatively, phage *C *may be produced by a recombination of *A *and *D*:

$$\begin{array}{l} \underline{\begin{array}{lllllll} A= & 2 & 1 & 4 & 3 & 2 & 1\\ B= & 2 & 2 & 4 & 2 & 2 & 1 \end{array}}\\ \begin{array}{lllllll} C= & 2 & 2 & 4 & 3 & 2 & 1 \end{array} \end{array}$$

Throughout the paper, a parent/child relationship is written with an arrow so that *A *→ *B *implies that *A *is a possible parent of *B*. We say that *C *is a *potential child *of *A *and *B*, written *A *→ *C *← *B*, or of *A *and *D*, written *A *→ *C *← *D*. A *recombination history *is simply a set of recombinations.

The first problem that we study in this paper is the following:

**Problem 1**. *Given a set  P of phages, reconstruct a *recombination history *such that a maximum number of phages in  P are produced by recombinations of elements of * P.

Reconstruction of recombination histories may be hampered by missing data: the number of sequenced phages is growing, but it is difficult to know how well the phage populations are sampled. Our second problem is to develop techniques to solve the following:

**Problem 2**. *Given a set  P of phages, find a minimal set of missing parents  Q such that a maximum number of phages in  P are produced by recombinations of elements of *(P∪Q).

## Results and discussion

### Alignment of 31 Staphyloccocus aureus phage genomes

**Figure 2:**
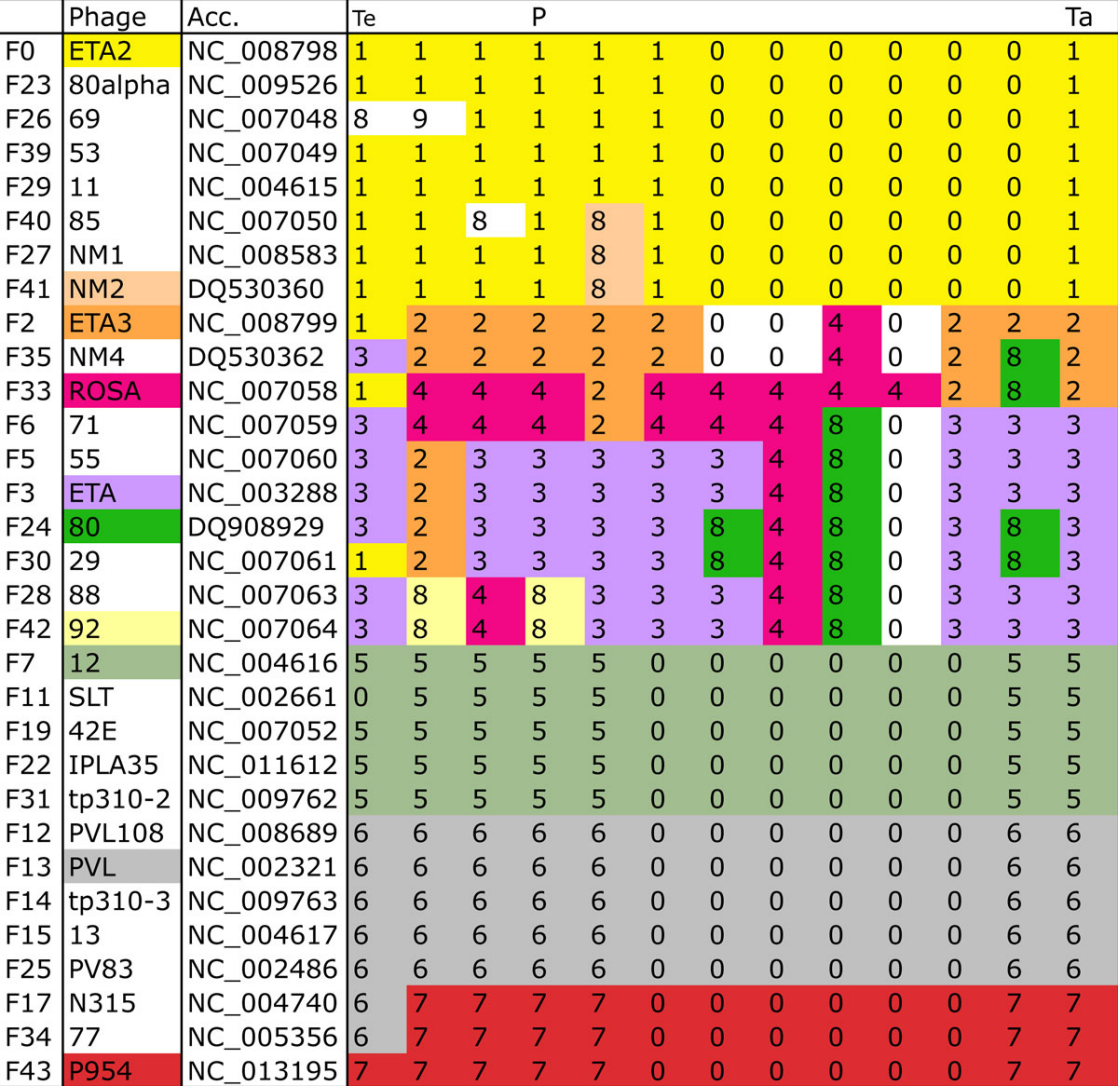
**Alignment fragment**. Fragment of an alignment of 31 *Staphyloccocus aureus *phage genomes that covers the segments between their terminase gene [module Te] and their tape measure gene [module Ta]. Module P is the anchor module corresponding to the portal gene. In a column, two cells that have the same color (or number) are highly similar. Empty sequences are designated by 0, and colored by their neighboring cells if both have the same color. Cells with variants that are unique are not colored.

We selected a set of 31 phage genomes having reasonably good annotations, possessing an individual accession number, and cataloged in a recent paper whose goal was to classify them [8]. Using a semi-automated technique (see Methods), we aligned the 31 sequences over approximately 80% of their length, including the complete subsequence spanning from the terminase genes to the amidase genes, which covers at least 50% of the genome sequences. Four other parts of the sequences were selected: the integrase genes, the repressor genes, part of the replication modules, and the *dut *gene. Figure 2 shows the modules and variants obtained from the alignment for the portion between the terminase genes and the tape measure genes. Additional file 1 contains the full alignment in detail.

The total number of modules is *k *= 38 and the number of non-empty variants ranges from 2 to 12. A surprising discovery is the presence of rather small - around 50 bp - highly conserved segments across all, or almost all, phages of the set. These occur at regular intervals along the first half of the sequences and seem to act as recombination *hubs *between larger modules. Details about their use is given in the next section.

Table 1: Quality of variants of the portal module: two sequences in variant *i *share at least *M *%, identity, and a sequence in variant *i *shares at most *m*% identity with a sequence in another variant.

**Table 1:** Quality of portal module variants

Variant	1	2	3	4	5	6	7	8
Length (bp)	1536	1425	1419	1425	1230	1326	1188	1425

*M *(%)	94	99	96	99	98	99	98	100

*m *(%)	54	51	64	76	54	51	61	76

Two sequences in variant *i *share at least *M *%, identity, and a sequence in variant *i *shares at most *m*% identity with a sequence in another variant.

Anchor modules correspond to well-annotated genes or regions. In the alignment of Figure 2, the portal and tape measure genes are anchors. The portal module is the most variable with 8 variants. A measure of quality of a module is how well the variants are separated, that is, how similar are sequences in the same variant, and how dissimilar are sequences in different variants. Table 1 gives these values for the 8 variants of the portal module. As one can see, two sequences in the same variant share at least 94% identity, and two sequences in different variants share at most 76% identity. The dataset extracted from the alignment is in Additional file 2.

### Modular recombination history

**Figure 3:**
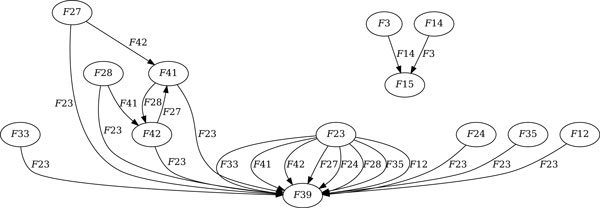
**The parents graph**. The parents graph for the set of 31 phages. Vertices that are not incident to at least one edge are omitted. Vertices are labeled by phage name. The edge labeled *F*41, coming from phage *F*28 and going to phage *F*42, indicates that *F*42 can be produced by a recombination between *F*28 and *F*41.

The first step in the reconstruction of the recombination history is to identify all *observable recombinations *consisting of two parents and their potential child: *P *→ *Q *← *R*. This detection step is easy, since we can simply check all trios for potential parent/child relationships. For the dataset of 31 phages we find 11 observable recombinations, summarized in Figure 3, involving four potential children. One of them, *F*15, has a clear set of parents, and phage *F*39 has *F*23 as a parent with a large number of other potential parents. The relationship between phages *F*41 and *F*42 is less clear and is the root of the complexity of the procedure: each of them can be a parent of the other, and each of them has only one possible set of parents. This yields two different solutions containing three recombination events, once the second parent of *F*39 is chosen. In general, cycles in the graph such as this, along with longer cycles, can have complex interactions that contribute to making the problem of removing cycles challenging.

**Figure 4:**
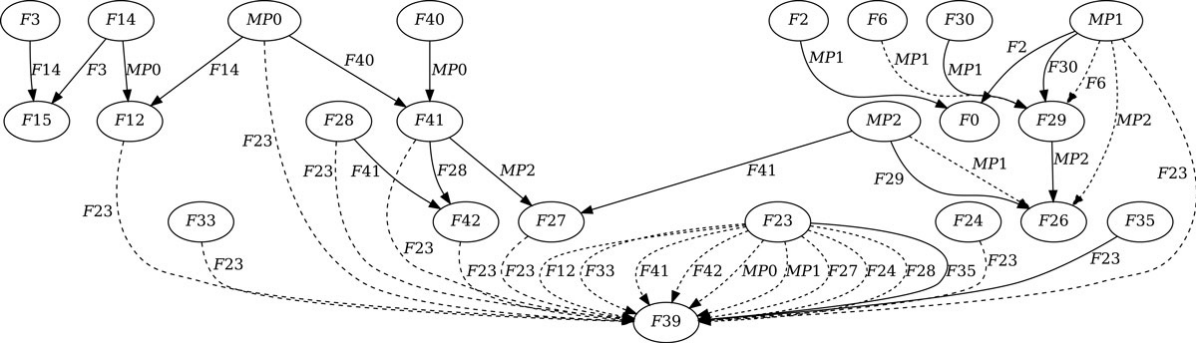
**A supergraph of the maximum recombination graph**. The solid edges depict a maximum recombination graph for the set of 31 phages, augmented by the three missing parents *M P*0, *M P*1, and *M P*2. The graph corresponds to a recombination history with nine recombinations. Alternative recombinations represented by dashed lines can be substituted for solid lines to form other maximum recombinations graphs.

However, there is always the possibility that some parents are not yet discovered, not yet sequenced, or even extinct. Using the techniques described in the Methods section, we constructed three 'virtual' missing parents that contribute to five more observable recombinations. The predicted modules of the missing parents are given in Additional file 2, and the resulting graph is presented in Figure 4.

The most interesting result is that this new solution solves the problem of who between *F*41 and *F*42 came first. Phage *F*40 appears as a parent of phage *F*41, thus freeing it to become the parent of *F*42. Such a combinatorial prediction can sometimes be confirmed at the sequence level. Indeed, phages that are involved in a sequence *P*_1 _→ *P*_2 _→ *P*_3 _may share inherited modules, and the phylogenetic history of those shared modules should be consistent with the prediction. In this case, phages *F*40, *F*41 and *F*42 share 6 such modules, and all of them confirmed the prediction.

Another interesting observation is the re-use of flanking *hub *modules in the predicted recombinations. Of course, the 9 recombinations require the use of a total of 18 flanking modules. Out of these 18 flanking modules, at least 8 belong to a set of 4 short modules: {*a*_2_, *a*_4_, *a*_9_, *a*_13_} - numbers refer to the columns of the full alignment given in Additional file 1. This means that at most 10 of the flanking modules belong to the remaining set of 34 modules.

## Methods

### Phage genome alignments

The alignment technique relies on two basic constructions: blocks and anchor modules.

For constants *m *and *M*, a *block  B*is an alignment of ℓ subsequences *b*_1_, *b*_2_, . . . , *b_ℓ _*from a set of phages * P*such that:

1. Each phage in  P has at most one subsequence in  B.

2. The identity between two sequences in  B is greater than *M *%.

3. The identity between a sequence in  B and any other subsequence not in  B (but in  P) is smaller than *m*%.

The requirements for blocks are generally hard to meet for most sets of genomes, but are rather common in bacteriophages, often with values of *M *larger than 90, together with values of *m *smaller than 70, as shown in [8].

An *anchor module *for a biological function *f *is a collection of blocks (each block corresponding to a variant) where each block of the collection should have a sequence annotated with function *f*. We have:

**Proposition 1**. *If the genomes in * P*are co-linear, then the anchors can be linearly ordered such that the first coordinates of all corresponding subsequences in a phage P are increasing*.

It thus makes sense to consider the set of non-empty sequences *S *= {*s*_1_, *s*_2_, . . . , *s_n′_*}, *n*' ≤ *n *that links two anchors. The next step in the alignment is to construct a set of *compatible *blocks over *S*:

**Definition 2**. *Two blocks are *compatible *if no sequence in one block is a subsequence of a sequence in the other one*.

A set  C of compatible blocks is partially ordered by the first coordinates of the subsequences of the phages that two blocks have in common. Thus, given a linear extension of the partial order, a sequence of modules containing one block each can always be defined. [In practice, consecutive modules that have no common phages can always be merged.]

The quality of an anchor module is assessed by the differences *M *− *m *for each block it contains. The quality of an alignment between two anchor modules is assessed by the differences *M *− *m *for each block, and the percentage of the nucleotide positions of the sequences in *S *included in the blocks.

### Reconstruction of the recombination history

Given a set  P of phages, the set  C of *potential children *is the subset of phages in  P that can be produced by at least one recombination of two phages in  P, called *observable recombinations*. A *parents graph *is a directed graph with vertices  P and two edges for each possible observable recombination, one from each parent to the child. Figure 3 depicts a parents graph. A *recombination graph *is a subgraph of the parents graph that is acyclic, where each node has either two incoming edges, its parents, or none. A *full *graph is a recombination graph in which no recombination can be added without violating the acyclic condition. A *maximum *recombination graph is a graph that contains a maximum number of recombinations, and is clearly full. Figure 4 depicts a maximum recombination graph with solid edges.

In a recombination graph, the subset  F of *forefathers *is the set of vertices that have one or more outgoing edges, but no incoming edge. The subset  D of *descendants *is the set of vertices that have two incoming edges.

There is a simple algorithm that constructs a full recombination graph subject to the constraint that its set of forefathers is included in a fixed subset S⊆P:

Algorithm 1 is the greedy answer to a maximization problem that is the first step of the solution of Problem 1. We first need a suitable family of feasible solutions that will be shown to form a matroid.

**Definition 3**. *Let * P*be a set of phages, with potential children * C. *A subset * E*of * C*is *feasible *if there exists a recombination graph with forefathers *F⊆(P\C)*such that *E⊆D.

**Proposition 2**. *The family of subsets of * C*formed by feasible subsets is a matroid*.

**Algorithm 1 **Construct a full graph with forefathers in  S

**Input: **list  L of observable recombinations and set of potential forefathers *S*

**Output: **a full recombination graph  G whose forefathers are in *S*

1: $S^{\prime }:=S$

2: **repeat**

3:      **for **each recombination *P *→ *Q *← *R *in  L**do**

4:        **if ***P, R *∈ *S*' and *Q **S*' **then**

5:          Add *P *→ *Q *← *R *to  G

6:          Add *Q *to $S^{\prime }$

7:        **end if**

8:      **end for**

9: **until **nothing is added to  G

*Proof*. Clearly, any subset  E of a feasible solution $E^{\prime }$ is feasible. Suppose that $|E|\;<\;|E^{\prime }|\;$, let  G be a recombination graph with children  E and descendants  D, and $G^{\prime }$ be a recombination graph with children $E^{\prime }$ and descendants $D^{\prime }$. Pick one vertex *X*_0 _in $E^{\prime }\backslash E\;$. We construct a sequence *X*_0 _. . . *X_k_*, such that *X_k _*is in $E^{\prime }\backslash E\;$, and *X_k _*has two parents in D∪F. If *X*_0 _has two parents in D∪F, then we are done and *k *= 0. If *X_i _*is in *ε*'\ *ε *and has at least one parent outside D∪F, choose one parent to be *X_i+1_*. Eventually the process must stop, since all descendants of a recombination graph have at least one ancestor with both parents in  F. Phage *X_k _*can be added to  E without creating a cycle.

**Corrollary 1**. *Algorithm 1 computes a maximum solution to Problem 1 with the constraint that the forefathers are in *P\C.

We now turn to the problem of constructing a maximum recombination graph. Clearly, the set of descendants  D is included in  C, thus |C| is an upper bound for the maximum number of phages produced by recombinations. The converse is not always true, but almost, in the sense that all potential children in  C are necessarily members (as parent, or child, or both) of any full recombination graph. We call it the *no-child-left-behind *property:

**Lemma 1 **(no-child-left-behind). *If a recombination graph for a set of phages * P*is full, then the set of potential children * C*is included in*D∪F, *the union of the forefathers and descendants of the recombination graph*.

*Proof*. Suppose that Q∈C is not in D∪F. Then *Q *has no child in the graph, and there exist parents *P *and *R *such that *P *→ *Q *← *R*. So adding the edges from *P *to *Q *and *R *to *Q *cannot create a cycle, which is a contradiction to the fullness of the graph.

The main consequence of Lemma 1 is to redefine the optimization goal of Problem 1 from maximizing |D| to minimizing |C∩F|, since the set * C*is fixed. At this point, we strongly suspect that the general problem is NP-complete. However, as we will see in the next sections, with proper optimization the most complex biological data that we could find yielded instances of size up to 5, which can easily be solved by exhaustive search. The optimization step is a consequence of the following proposition that extend easily computed partial solutions to a maximum solution.

**Proposition 3**. *Any full recombination graph whose forefathers are in *P\C*is either a maximum recombination graph, or can have recombinations added to it to become maximum*.

*Proof*. Let  G be a full graph with descendants  D, whose forefathers  F are in P\C, and let  M be a maximum graph with descendants $D^{\prime }$ and forefathers $F^{\prime }$. Consider the acyclic subgraph of $M^{\prime }$ of  M induced by the forefathers $F^{\prime }\backslash F$, and the vertices $D^{\prime }\backslash D$. By construction, the vertices and edges of  G and $M^{\prime }$ are disjoint. Any vertex of $D^{\prime }\backslash D$ is connected, in $M^{\prime }$, to at least one parent, otherwise this would contradict the fullness of  G. For vertices that have only one parent in $M^{\prime }$, the other one must be in  G. Therefore all the missing arcs go from vertices of  G to vertices of $M^{\prime }$, and can be added together without creating cycles.

Combining these results, Problem 1 can be solved with the following steps:

1. Compute a full recombination graph  G using Algorithm 1 and the set P\C.

2. Let  B be the subset of potential children  C that are not in  G. Find a minimum subset $F^{\prime }$ of  B such that all elements of $B\backslash F^{\prime }$ have parents in  B,  G, or P\C.

Lemma 1 guarantees that step 2 has a solution, and Proposition 3 guarantees that the procedure will give a maximum solution.

### Missing parents

We propose here a technique for reconstructing missing parents that is based on the following observation:

**Proposition 4**. *The recombination children of phages P and Q share at least three consecutive modules with each parent*.

Suppose phages *P *and *Q *share at least three consecutive modules then phage *P may *be a parent of phage *Q*, or phage *Q may *be a parent of phage *P *. Those two hypotheses will be denoted *P *→ *Q *and *Q *→ *P *, or simply *P *↔ *Q*. A *reconstruction history *must decide, for each pair of phages that share at least three consecutive modules, whether *P *→ *Q, Q *→ *P *, or neither.

When *P *→ *Q*, it is possible to partially identify the *missing parent(s)*. Let *P = abxcdy*, and *Q = a*'*bxcd*'*y*' be a decomposition of the sequences in which *bxc *is a maximal sequence of at least three consecutive shared modules between *P *and *Q*: that is, modules *a *and *a' *are different, modules *d *and *d' *are different, *x *is a sequence of at least one module. Then, if *P *→ *Q*, the missing parent can be written as *P_Q _= a'b *∗ .. ∗ *cd'y'*, where the ∗..∗ represents any sequence of modules of length |*x*|, but different from *x*. [Note that *P *and *Q *may share two or more groups of at least three consecutive modules, separated by non-shared modules, yielding alternative missing parents.]

For example, consider the following set of phages, that contains only one observable recombination *A *→ *C *← *D*:

$$\begin{array}{ccccccc} A= & 2 & 1 & 4 & 3 & 2 & 1\\ C= & 2 & 2 & 4 & 3 & 2 & 1\\ D= & 2 & 2 & 4 & 2 & 2 & 1\\ G= & 1 & 2 & 3 & 1 & 1 & 2\\ H= & 2 & 2 & 3 & 1 & 1 & 2 \end{array}$$

We have: *A *↔ *C, A *↔ *D, C *↔ *D*, and *G *↔ *H*. These relations give 8 *templates *for missing parents. Here are the templates for the relations *G *↔ *H*:

$$\begin{array}{c} G\to H\leftarrow G_{H}=2\;2***2\\ H\to G\leftarrow H_{G}=1\;2***2 \end{array}$$

**Figure 5:**
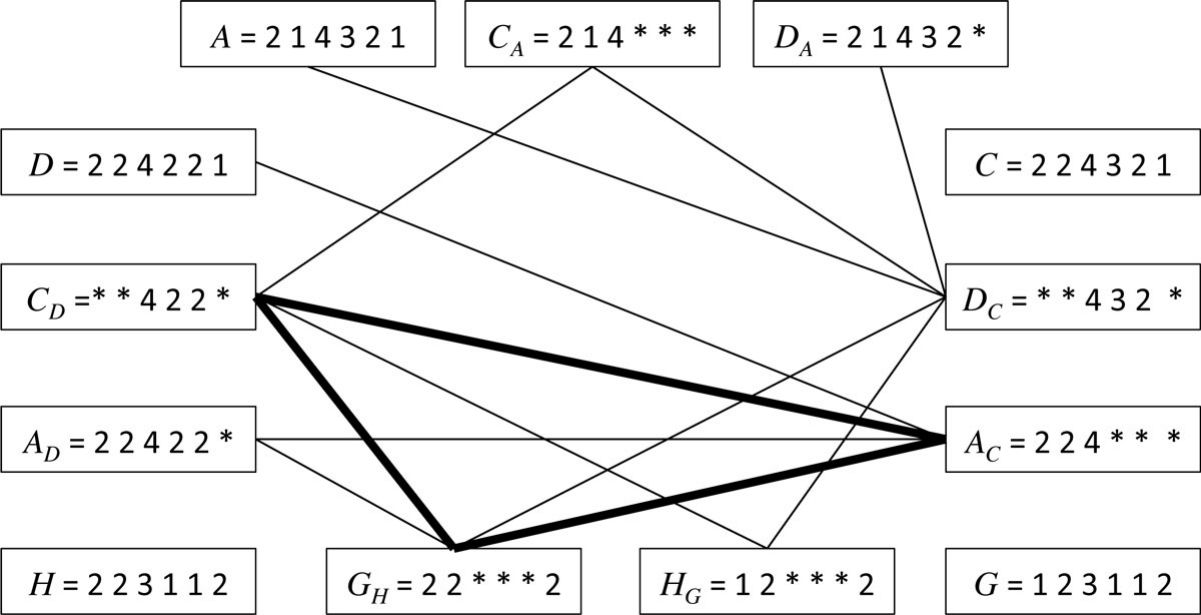
**The missing parents graph**. The clique in bold lines contains only templates, leading to missing parent 2 2 4 2 2 2.

Two or more templates are *compatible *if the sets of phages that they represent have a non-empty intersection. [Note that alternative parents are never compatible.] The *missing parents graph *is the graph of the compatibility relation over a set of phages and all the missing parent templates deduced from the set. Figure 5 shows the corresponding graph for the above example. In this example, the edge exists between templates *C_A _*= 2 1 4 ∗ ∗ ∗ and *D_C _*= ∗ ∗ 4 3 2 ∗ since they are compatible as they do not specify conflicting variants for any module; 2 and '*' are compatible, 1 and '*' are compatible, 4 and 4 are compatible, and so on.

Cliques that contain an observed phage correspond to observable recombination events. The most interesting cliques are those whose elements are all templates: they describe missing parents. For example, in Figure 5, the clique that contains *G_H _*= 2 2 ∗ ∗ ∗ 2, *C_D _*= ∗ ∗ 4 2 2 *∗ *and *A_C _*= 2 2 4 ∗ ∗ ∗ spells out phage 2 2 4 2 2 2, which is the only phage that belongs to all sets represented by the three template vertices of the clique. Opting for this solution, we would infer one missing phage *B *= 2 2 4 2 2 2, and three recombination events: *A *→ *C *← *B, C *→ *D *← *B*, and *G *→ *H *← *B*.

How to add missing parents

Adding missing parents must be done carefully in order to insure that the procedure converges if applied recursively. For the case of the data in the Results section, we used the following guidelines:

1. A missing parent must not be a potential child of the set of phages to which it is added.

2. It must increase the total number of recombinations by at least two.

3. Missing parents of orphans, these being the members of *C *that have no parents in the recombination graph, have priority.

Selecting an optimal choice of missing parents could be, in theory, a difficult problem. Since it was not the case in practice, we postpone the theoretical analysis to a subsequent paper. It will also be worthwhile to study the properties of the set of all optimal solutions, especially in terms of what is common to all optimal solutions.

## Conclusions

This paper provides, to the best of our knowledge, the first formal attempt to reconstruct the modular recombination histories of families of phages. The results are encouraging, but raise many questions and point out many directions for further development.

Our next goal is to apply these techniques to the whole family of phages that co-infect *Staphylococcus aureus*, currently thought to contain more than a hundred members. This will be computationally intensive, but our preliminary studies suggest that this will be feasible.

Since *Staphylococcus aureus *bacteria are the source of many infections in human beings, strains have been collected in hospitals and other sites all over the world. Another direction would link the recombination histories to geographical data.

Other important phage families are also under special scrutiny, for example the phages that infect *Lactococcus lactis *bacteria that are essential in the production of cheese [9] and whose presence can lead to taste alteration or complete fermentation failure.

## Competing interests

The authors declare that they have no competing interests.

## Supplementary Material

Additional file 1 **The complete alignment**. This file contains the complete alignment as described in the Results section. Click here for file

Additional file 2 **The dataset extracted from the alignment**. The variants and modules obtained from our alignments, along with missing parents computed using our methods. Click here for file

## Declarations

This work was partially supported by NSERC grant 121768-2009.

This article has been published as part of BMC Bioinformatics Volume 14 Supplement 15, 2013: Proceedings from the Eleventh Annual Research in Computational Molecular Biology (RECOMB) Satellite Workshop on Comparative Genomics. The full contents of the supplement are available online at http://www.biomedcentral.com/bmcbioinformatics/supplements/14/S15.
