# Supplementary material for: Reconstructing the modular recombination history of Staphylococcus aureus phages
Source: BMC Bioinformatics. 2013 Oct 15;14(Suppl 15):S17. doi: 10.1186/1471-2105-14-S15-S17 (PMC3852002; doi:10.1186/1471-2105-14-S15-S17)
Supplement: Additional file 2 — The dataset extracted from the alignment. The variants and modules obtained from our alignments, along with missing parents computed using our methods. [file 1471-2105-14-S15-S17-S2.PDF]

$F0$	1	1	1	1	0	1	1	1	2	1	1	1	1	1	1	1	1	1	1	0	0	0	0	0	0	1	0	0	1	1	1	1	1	0	1	1	0	1
$F2$	1	1	1	1	0	1	1	1	2	2	0	0	1	1	2	2	2	2	2	0	0	1	0	1	1	2	0	0	2	2	1	1	2	0	1	1	0	1
$F3$	1	1	2	1	0	1	1	1	2	3	3	0	1	3	2	3	3	3	3	1	4	2	0	2	2	3	0	0	2	2	1	1	2	0	1	1	0	1
$F5$	1	1	9	1	0	3	3	8	2	2	0	0	1	3	2	3	3	3	3	1	4	2	0	2	2	3	0	0	2	2	1	1	2	0	1	4	0	2
$F6$	1	1	3	1	8	7	0	0	2	1	6	1	1	3	4	4	4	2	4	2	4	2	0	2	2	3	0	0	2	2	1	1	2	0	1	1	0	1
$F7$	2	2	2	2	0	2	0	0	2	1	5	1	1	5	5	5	5	0	0	0	0	0	0	0	4	0	0	0	0	0	1	0	0	0	0	0	0	3
$F11$	2	10	3	1	0	1	1	1	2	1	7	1	1	0	5	5	5	0	0	0	0	0	0	0	4	0	0	0	0	0	1	0	0	0	0	0	0	3
$F12$	2	1	4	1	4	4	1	1	2	1	7	1	1	9	6	6	6	0	0	0	0	0	0	0	5	1	1	3	4	3	0	9	9	0	2	0	3
$F13$	2	2	4	0	0	6	6	6	2	1	6	1	1	9	6	6	6	0	0	0	0	0	0	0	5	1	1	3	4	3	0	4	7	0	2	0	3
$F14$	3	1	4	1	9	0	3	3	2	1	7	1	1	9	6	6	6	0	0	0	0	0	0	0	5	1	1	3	4	3	0	4	7	2	3	2	4
$F15$	3	1	4	1	0	1	1	1	2	3	3	0	1	9	6	6	6	0	0	0	0	0	0	0	5	1	1	3	4	3	0	4	7	2	3	2	4
$F17$	3	1	2	3	0	6	8	6	2	2	0	0	1	9	7	7	7	0	0	0	0	0	0	0	6	1	2	4	4	3	0	9	9	0	2	2	4
$F19$	3	9	4	1	0	7	0	0	2	1	6	1	1	5	5	5	5	0	0	0	0	0	0	0	4	0	0	0	0	0	1	0	0	0	0	2	4
$F22$	4	0	5	2	0	2	0	0	9	9	0	0	1	5	5	5	5	0	0	0	0	0	0	0	4	0	0	0	0	0	1	0	0	0	0	0	3
$F23$	7	1	4	1	4	1	1	1	2	1	8	0	1	1	1	1	1	1	1	0	0	0	0	0	0	1	0	0	1	1	1	1	1	0	1	1	0	3
$F24$	6	1	6	1	4	4	1	1	2	1	7	1	1	3	2	3	3	3	3	3	4	2	0	2	3	3	0	0	2	2	1	1	2	0	1	1	0	2
$F25$	5	1	7	1	0	3	3	3	2	2	0	0	1	9	6	6	6	0	0	0	0	0	0	0	5	1	1	3	4	3	0	4	7	2	3	4	4
$F26$	5	1	4	1	0	1	1	1	2	3	10	0	1	8	9	1	1	1	1	0	0	0	0	0	0	1	0	0	1	1	1	1	1	0	1	1	0	2
$F27$	5	1	8	1	4	4	1	1	2	3	3	0	1	1	1	1	1	8	1	0	0	0	0	0	0	1	0	0	1	1	1	1	1	0	1	1	0	3
$F28$	5	1	10	1	4	4	1	1	2	9	0	0	1	3	8	4	8	3	3	1	4	2	0	2	2	3	0	0	2	2	1	1	2	0	1	1	0	2
$F29$	5	1	4	1	8	7	0	0	2	1	6	1	1	1	1	1	1	1	1	0	0	0	0	0	0	1	0	0	1	1	1	1	1	0	1	1	0	2
$F30$	5	1	8	1	8	7	0	0	2	1	6	1	1	1	2	3	3	3	3	3	4	2	0	2	3	3	0	0	2	2	1	1	2	0	1	1	0	2
$F31$	6	2	4	2	0	2	0	0	2	1	7	0	1	5	5	5	5	0	0	0	0	0	0	0	4	0	0	0	0	0	1	0	0	0	0	0	3
$F33$	6	1	8	1	4	4	1	1	2	1	4	1	1	1	4	4	4	2	4	2	4	1	4	1	3	2	0	0	1	2	1	1	2	0	1	1	0	3
$F34$	6	1	4	1	4	8	1	1	2	1	5	1	1	9	7	7	7	0	0	0	0	0	0	0	6	2	2	4	4	3	0	4	7	2	3	0	3
$F35$	6	1	3	1	4	4	1	1	2	3	3	0	1	3	2	2	2	2	2	0	0	1	0	1	3	2	0	0	2	2	1	1	1	0	1	1	0	3
$F39$	7	1	4	1	4	4	1	1	2	1	8	0	1	1	1	1	1	1	1	0	0	0	0	0	0	1	0	0	1	1	1	1	1	0	1	1	0	3
$F40$	7	1	6	1	4	8	1	1	2	1	5	1	1	1	1	8	1	8	1	0	0	0	0	0	0	1	0	0	1	1	1	1	1	0	1	1	0	2
$F41$	7	1	4	1	4	4	1	1	2	3	3	0	1	1	1	1	1	8	1	0	0	0	0	0	0	1	0	0	1	1	1	1	1	0	1	1	0	2
$F42$	7	1	4	1	4	4	1	1	2	9	0	0	1	3	8	4	8	3	3	1	4	2	0	2	2	3	0	0	2	2	1	1	2	0	1	1	0	2
$F43$	7	1	7	1	0	7	0	0	2	1	4	1	1	7	7	7	7	0	0	0	0	0	0	0	6	2	2	4	4	3	0	4	7	2	3	2	4
$MP0$	2	1	4	1	4	4	1	1	2	3	3	0	1	1	1	1	1	*	*	*	*	*	*	*	*	*	*	*	*	*	3	0	9	9	0	2	0	3
$MP1$	5	1	4	1	4	4	1	*	2	1	1	1	1	1	1	1	1	1	1	0	0	0	0	0	0	1	0	0	1	1	1	1	1	0	1	1	0	2
$MP2$	5	1	8	1	0	1	1	1	2	3	10	0	1	8	9	1	*	*	*	*	*	*	*	*	*	*	*	*	*	*	*	*	*	*	*	1	0	3
