# Supplementary material for: Reconstructing the modular recombination history of Staphylococcus aureus phages
Source: BMC Bioinformatics. 2013 Oct 15;14(Suppl 15):S17. doi: 10.1186/1471-2105-14-S15-S17 (PMC3852002; doi:10.1186/1471-2105-14-S15-S17)
Supplement: Additional file 1 — The complete alignment. This file contains the complete alignment as described in the Results section. [file 1471-2105-14-S15-S17-S1.PDF]

	Name	Accession	Integrase	hub1	Repressor	hub2	Replication module				hub3	Dut			hub4	Terminase			Portal									Tape												Holin_Amidase
			1 963 99%<br>2 1206 98%<br>3 1037 90%<br>4 1248 100%<br>5 959 98%<br>6 1187 96%<br>7 1385 98%	1 51 98%<br>2 81 96%<br>9 418 100%<br>10 100 100%	1 1770 100%<br>2 402 90%<br>3 213 99%<br>4 409 99%<br>5 606 100%<br>6 587 95%<br>7 400 98%<br>8 446 99%<br>9 1455 100%<br>10 599 100%	1 222 90%<br>2 981 96%<br>3 To be resolved	4 991 99%<br>8 497 94%<br>9 890 100%	1 473 99%<br>2 3372 98%<br>3 190 98%<br>4 483 99%<br>6 2230 96%<br>7 1842 97%<br>8 458 100%	1 294 92%<br>3 249 97%<br>6 707 100%<br>8 709 100%	1 844 97%<br>3 838 96%<br>6 2507 98%<br>8 874 100%	2 229 90%<br>9 886 100%	1 291 95%<br>2 532 96%<br>3 254 94%	1 125 100%<br>3 294 94%<br>4 125 99%<br>5 137 100%<br>6 106 99%<br>7 122 100%<br>8 225 100%<br>9 334 96%<br>10 264 100%	1 118 97%	1 46 97%	1 558 92%<br>3 465 96%<br>5 1961 95%<br>7 497 100%<br>8 601 100%<br>9 227 99%	1 160 97%<br>2 702 90%<br>4 423 94%<br>5 1307 92%<br>6 1469 99%<br>7 997 99%<br>8 608 100%<br>9 To be resolved	0 1796 100%<br>1 1707 99%<br>2 1286 99%<br>3 1255 97%<br>4 505 94%<br>5 2226 94%<br>6 2415 99%<br>7 2122 98%<br>8 1795 100%	1 2839 95%<br>2 1407 99%<br>3 1434 97%<br>4 2177 99%<br>5 6308 99%<br>6 6476 99%<br>7 3876 98%<br>8 2288 100%	1 278 97%<br>2 928 92%<br>3 929 96%<br>8 294 99%	1 4488 98%<br>2 2361 99%<br>3 585 92%<br>4 603 100%	1 941 97%<br>2 807 100%<br>3 995 99%	4 671 91%	1 551 99%<br>2 341 96%	4 966 100%	1 2276 100%<br>2 2494 98%	1 209 100%<br>2 300 99%<br>3 469 98%	1 5696 96%<br>2 4700 99%<br>3 4692 96%<br>4 12760 97%<br>5 10838 98%<br>6 4449 96%	1 1318 95%<br>2 1327 95%	1 891 99%<br>2 797 94%	1 634 93%<br>2 636 93%<br>3 787 97%	1 200 90 %<br>2 200 91%<br>4 889 99%	1 3991 89%<br>3 2601 96%	1 553 86 %<br>Split?	1 1275 94%<br>2 1251 97 %<br>4 195 93%<br>9 194 99%	7 1679 98%<br>9 1678 99%	1 1659 94%<br>2 122 97 %	1 534 95%<br>2 588 98%<br>3 391 93%<br>4 533 100%	2 397 93 %<br>4 395 100%	1 1674 95 %<br>2 1863 90%<br>3 1432 92%<br>4 1020 94%
F0	ETA2	NC_008798	1 (53..1016) *	1 (1111..1161) *	1 (2295..4065) *	1 (9088..9309)*	0	1 (9748..10220)*	1 (10220..10514)*	1 (10523..11366)*	2 (13216..13445) 90%	1 (14131..14426)*	1 (14426..14550)*	1 (14553..14670)*	1 14670	1 (15508..16065)*	1 (16072..16231)*	1 (16231..17937) *	1 (17937..20776)*	1 (20776..21054)*	1 (21054....25542)*	0	0	0	0	0	0	1 (25542..31238)*	0	0	1 (31238..31874)*	1 (31874..32095)*	1 (32095..36085)*	1 (36085..36637)*	1 (36637..37911)*	0	1 (37911..39569)*	1 (39730..40263)*	0	1 (40263..41937)*
F2	ETA3	NC_008799	1 (53..1016) 99%	1 (1113..1158) 100%	1 (2294..4064) 100%	1 9145	0	1 (9805..10277) 100%	1 (10277..10571) 100	1 (10580..11423) 99%	2 (13219..13448) *	2 (14722..15253)*	0	0	1 15253	1 (16300..16857) 94%	2 (16878..17579)*	2 (17579..18864)*	2 (18864..20271)*	2 (20271..21199)*	2 (21199....23560)*	0	0	4 (23560....24111) 99%	0	2 (24108..26384)*	2 (26384..26593)*	2 (26593..31293) *	0	0	2 (31293..31929) *	2 (31937..32136) 91%	1 (32136..36126) 92%	1 (36126..36678) 98%	2 (36678..37928) 97%	0	1 (37928..39586) 95%	1 (39747..40280) 99%	0	1 (40280..41954) 98%
F3	ETA	NC_003288	1 (53..1016) 99%	1 (1111..1158) 98%	2 (2342..2744) *	1 9230	0	1 (10302..10774) 99%	1 (10774..11068) 99%	1 (11077..11921) 95%	2 (13640..13866) 93%	3 (14927..15180)*	3 (15180..15473)*	0	1 15473	3 (16277..16741)*	2 (16752..17450) 90%	3 (17450..18704)*	3 (18704..20138) *	3 (20138..21067) *	3 (21067..21652) *	3 (21652..22593)*	4 (22593..23264) 96%	8 (23264..23605) 96%	0	3 (23605..26099) *	3 (26099..26399)*	3 (26399..31091)*	0	0	2 (31091..31727) 99%	2 (31735..31934) 99%	1 (31934..35924) 91%	1 (35924..36477) 92%	2 (36477..37727) 97%	0	1 (37727..39385) 95%	1 (39546..40079) 99%	0	1 (40079..41753) 97%
F5	55	NC_007060	1 (27182..28145) 99%	1 (28240..28288) 98%	9 (29051..30506)*	1 35720	0	3 (36258..36447) 98%	3 (36447..36695) 96%	8 (36695..37568)*	2 (38943..39172) 97%	2 (40100..40631) 98%	0	0	1 40631	3 (41294..41758) 97%	2 (41769..565) 89-97%	3 (565..1819) 99%	3 (1819..3253) 99%	3 (3253..4182) 97%	3 (4182..4767) 99%	3 (4767..5708) 97%	4 (5708..6379) 96%	8 (6379..6720) 98 %	0	3 (6720..9214) 99%	3 (9214..9514) 99%	3 (9514..14206) 97%	0	0	2 (14212..14839) 92%	2 (14847..15046) 98%	1 (15046..19036) 93%	1 (19036..19588) 98%	2 (19588..20838) 97%	0	1 (20838..22496) 94%	4 (22657..23189) *	0	2 (23189..25052) 92%
F6	71	NC_007059	1 (27167..28130) 99%	1 (28225..28272) 98%	3 (29653..29866) 99%	1 35503	8 (36041..36537)*	7 (36537..38318) 97%	0	0	2 (39534..39763) 97%	1 (40848..41137) 96%	6 (41137..41242) 99%	1 (41242..41358) 98%	1 41358	3 (42434..42898) 99%	4 (1..423) 94%	4 (423..926) 95%	4 (926..3103) 99%	2 (3103..4029) 92%	4 (4029 ..4632) 100%	4 (4632..5574) 100%	4 (5574..6245) 100%	8 (6245..6586) 96%	0	3 (6586..9080) 98%	3 (9080..9380) 99%	3 (9380..14072) 96%	0	0	2 (14078..14708) 97%	2 (14716..14915) 100	1 (14915..18904) 90%	1 (18904..19457) 97%	2 (19457..20838) 97%	0	1 (20707..22365) 94%	1 (22460..22992) 97%	0	1 (22992..24660) 95%
F7	12	NC_004616	2 (84..1290) *	2 (1290..1370)*	2 (3891..4292) 91%	2 (7023..8023)*	0	2 (8023..11394)*	0	0	2 (11964..12187) 92%	1 (13689..13979) 98%	5 (13979..14115)*	1 (14115..14232) 98%	1 14232	5 (17270..19230)*	5 (19230..20536)*	5 (20548..22773)*	5 (22773..29081)*	0	0	0	0	0	0	0	0	5 (29081..41841) *	0	0	0	0	0	1 (41841..42387) 87%	0	0	0	0	0	3 (42406..43836) 98%
F11	SLT	NC_002661	2 (113..1319) 99%	10 (1319..1419)*	3 (3742..3955) *	1 9626	0	1 (10286..10758) 100	1 (10758..11052) 100	1 (11061..11904) 99%	2 (13033..13262) 94%	1 (13513..13803) 97%	7 (13803..13924) 100	1 (13924..14041) 100	1 14041	0	5 (15419..16734) 92%	5 (16746..18972) 96%	5 (18972..25280) 99%	0	0	0	0	0	0	0	0	5 (25280..38044) 98%	0	0	0	0	0	1 (38044..38591) 86%	0	0	0	0	0	3 (38610..40038) 93%
F12	PVL108	NC_008689	2 (383..1588) 98%	1 (1595..1642) 100%	4 (3434..3834) *	1 10366	4 (10565..11555) 100	4 (11555..12037) 100	1 (12037..12331) 97%	1 (12340..13183) 97%	2 (14568..14797) 95%	1 (15406..15696) 97%	7 (15696..15817)*	1 (15817..15934)*	1 15934	6 (16613..16839)*	6 (16839..18288) 99%	6 (18280..20730) 99%	6 (20730..27206) 99%	0	0	0	0	0	0	0	0	6 (27206..29314) 99	1 (29314..30641) 95%	1 (30641..31532) 99%	3 (31532..32221) 99%	4 (32221..33109) 99%	3 (33109..35709) 98%	0	9 (35710..35903)*	9 (35903..37580) *	0	2 (37575..38162) 98%	0	3 (38181..39611) 99%
F13	PVL	NC_002321	2 (24300..25506) 99%	2 (25506..25586) 98%	4 (26588..26988) 99%	0	0	6 (30818..33046)*	6 (33046..33753)*	6 (33753..36259)*	2 (37085..37314) 96%	1 (37591..37880) 97%	6 (37880..37985)*	1 (37985..38101) 98%	1 38104	6 (39707..39933) 99%	6 (39933..41401)*	6 (1..2451)*	6 (2451..8926)*	0	0	0	0	0	0	0	0	6 (8926..11032)*	1 (11032..12350)*	1 (12350..13240) *	3 (13240..13928)*	4 (13928..14816) 99%	3 (14816..17415) 98%	0	4 (17415..17608) 99%	7 (17608..19285) 98%	0	2 (19280..19867)*	0	3 (19886..21316) *
F14	tp310-3	NC_009763	3 (58..1095) *	1 (1100..1146) 98%	4 (2251..2651) 99%	1 8495	9 (9206..10095)*	0	3 (10095..10343) 96%	3 (10343..11180) 96%	2 (12565..12794) 95%	1 (13403..13693) 97%	7 (13693..13814) 100	1 (13814..13931) 100	1 13931	6 (14610..14836) 99%	6 (14836..16285) 99%	6 (16277..18727) 99%	6 (18727..25203) 99%	0	0	0	0	0	0	0	0	6 (25203..27310) 99%	1 (27310..28637) 95%	1 (28637..29528) 99%	3 (29528..30217) 99%	4 (30217..31105) 99%	3 (31105..33705) 99%	0	4 (33705..33898) 99%	7 (933898..35576) 99	2 (35576..35697) 100	3 (35697..36089) 98%	2 (36119..36515) 99%	4 (36515..37529) 95%
F15	13	NC_004617	3 (59..1096) 100%	1 (1101..1147) 100%	4 (2378..2778) 100%	1 9141	0	1 (9795..10267) 100%	1 (10267..10561) 100	1 (10570..11413) 100%	2 (12801..13024) 95%	3 (14204..14457) 94%	3 (14459..14750) 97%	0	1 14750	6 (15332..15558) 100	6 (15558..17026) 99%	6 (17018..19468) 99%	6 (19468..25944) 99%	0	0	0	0	0	0	0	0	6 (25944..28051) 99%	1 (28051..29378) 95%	1 (29378..30269) 99%	3 (30269..30958) 99%	4 (30958..31846) 99%	3 (31846..34446) 99%	0	4 (34446..34640) 100	7 (34640..36318) 99%	2 (36318..36439) 100	3 (36439..36829) 100	2 (36859..37255) 100	4 (37255..38269) 95%
F17	N315	NC_004740	3 (10..1047) 99%	1 (1052..1099) 98%	2 (4128..4529) 90%	3	0	6 (8372..10600) 96%	8 (10600..11308)*	6 (11308..13814) 98%	2 (14902..15128) 95%	2 (15386..15917) 96%	0	0	1 15917	6 (16551..16777) 99%	7 (16761..17757) 99%	7 (17757..19878) 98%	7 (19878..23754) 99%	0	0	0	0	0	0	0	0	7 (23754..28203) 96%	1 (28203..29527) 95%	2 (29536..30333) 94%	4 (30335..31122) 97%	4 (31122..32010) 99%	3 (32010..34610) 99%	0	9 (34611..34804) 99%	9 (34804..36481) 99%	0	2 (36476..37063) 99%	2 (38345..38741) 99%	4 (38741..39754) 95%
F19	42E	NC_007052	3 (27198..28235) 90%	9 (28235..28653)*	4 (29939..30339) 99%	1 35767	0	7 (36759..38540) 97%	0	0	2 (40039..40262) 97%	1 (40513..40802) 97%	6 (40802..40907) 100	1 (40907..41023) 98%	1 41023	5 (42593..44551) 97%	5 (44551..45860) 96%	5 (5..2230) 99%	5 (2230..8538) 100%	0	0	0	0	0	0	0	0	5 (8538..21298) 99%	0	0	0	0	0	1 (21298..21842) 86%	0	0	0	0	2 (21872..22258) 93%	4 (22258..23277) *
F22	IPLA35	NC_011612	4 (1..1248) *	0	5 (3579..4185) *	2 6105	0	2 (7105..10475) 98%	0	0	9 (10819..11705) *	9 (13277..13610) 96%	0	0	1 13610	5 (16678..18633) 97%	5 (18633..19944) 96%	5 (19956..22181) 94%	5 (22181..28489) 99%	0	0	0	0	0	0	0	0	5 (28489..41246) 97%	0	0	0	0	0	1 (41246..41792) 86%	0	0	0	0	0	3 (41811..43241) 96%
F23	80alpha	NC_009526	7 (32..1417) 99%	1 (1422..1469) 100%	4 (3185..3585) 99%	1 8178	4 (8380..9370) 100%	1 (9380..9852) 99%	1 (9852..10146) 100%	1 (10155..10998) 100%	2 (12365..12594) 94%	1 (13888..14177) 98%	8 (14177..14401) 100%	0	1 14401	1 (15694..16251) 99%	1 (16258..16416) 99%	1 (16416..18122) 99%	1 (18122..20962) 95%	1 (20962..21239) 97%	1 (21241..25728) 98%	0	0	0	0	0	0	1 (25728.31421) 96%	0	0	1 (31421..32057) 96%	1 (32057..32278) 100	1 (32278..36274) 90%	1 (36274..36827) 87%	1 (36827..38101) 96%	0	1 (38101..39759) 96%	1 (39854..40387) 99%	0	3 (40387..41818) 92%
F24	80	DQ908929	6 (25..1212) *	1 (1240..1285) 98%	6 (2901..3488) *	1 7689	4 (7888..8878) 100%	4 (8878..9360) 100%	1 (9360..9654) 97%	1 (9663..10506) 98%	2 (12312..12541) 94%	1 (13150..13440) 97%	7 (13440..13561) 100%	1 (13561..13678) 100	1 13678	3 (14386..14850) 97%	2 (14861..15561) 90%	3 (15563..16815) 97%	3 (16815..18249) 97%	3 (18249..19178) 96%	3 (19178..19763) 92%	8 (19763..20758)*	4 (20758..21429) 91%	8 (21429..21770) *	0	3 (21770..24269)* 98	8 (24269..24738)*	3 (24747..29430) 96%	0	0	2 (29436..30066) 97%	2 (30074..30273)*	1 (30273..34269) 89%	1 (34269..34822) 91%	2 (34822..36072)*	0	1 (36072..37730) 95%	1 (37891..38423) 98%	0	2 (38423..40286) 96%
F25	PV83	NC_002486	5 (94..1053) *	1 (1148..1196) 98%	7 (2923..3316) 98%	1 9250	0	3 (9788..9977)*	3 (9977..10225)*	3 (10225..11062)*	2 (12723..12946) 96%	2 (13480..14011) 96%	0	0	1 14011	6 (15073..15299) 99%	6 (15299..16758) 99%	6 (16750..19200) 99%	6 (19200..27601) 99%	0	0	0	0	0	0	0	0	6 (27601..29708) 99%	1 (29708..31032) 98%	1 (31032..31922) 99%	3 (31922..32611) 99%	4 (32611..33498) 99%	3 (33500..36099) 96%	0	4 (36099..36293) 93%	7 (36293..37971) 98%	2 (37971..38092) 100	3 (38092..38484) 93%	4 (38514..38909) *	4 (38909..39928) 94%
F26	69	NC_007048	5 (26829..27789) 98%	1 (27884..27931) 100%	4 (29406..29806) 99%	1 32253	0	1 (36080..36552) 99%	1 (36552..36846) 94%	1 (36855..37698) 98%	2 (39498..39727) 92%	3 (40132..40385) 94%	10 (40385..40648)*	0	1 40648	8 (42131..42732)* End	9	1 (109..1815) 99%	1 (1815..4655) 97%	1 (4655..4932) 98%	1 (4934..9421) 98%	0	0	0	0	0	0	1 (9421..15117) 97%	0	0	1 (15117..15753) 99%	1 (15753..15974) 99%	1 (15977..19964) 89%	1 (19964..20517) 86%	1 (20517..21791) 95%	0	1 (21791..23449) 98%	1 (23544..24076) 96%	0	2 (24076..25939) 95%
F27	NM1	NC_008583	5 (84..1044) 98%	1 (1139..1189) 100%	8 (3447..3892) 99%	1 8489	4 (8691..9681) 100%	4 (9681..10163) 100%	1 (10163..10457) 99%	1 (10466..11309) 99%	2 (12935..13158) 94%	3 (14712..14965) 95%	3 (14967..15258) 94%	0	1 15258	1 (15905..16462) 92%	1 (16469..16627) 97%	1 (16627..18333) 99%	1 (18333..21174) 96	8 (21173..21467) 100	1 (21471..25959) 99%	0	0	0	0	0	0	1 (25959..31655) 98%	0	0	1 (31655..32291) 100	1 (32291..32512) 100	1 (32512..36502) 92%	1 (36502..37055) 96%	1 (37055..38329) 96%	0	1 (38329..39987) 98%	1 (40082..40615) 96%	0	3 (40615..42046) 92%
F28	88	NC_007063	5 (26846..27806) 98%	1 (27901..27948) 100%	10 (29493..30092)*	1 34561	4 (34763..35753) 100	4 (35753..36235) 99%	1 (36235..36529) 97%	1 (36538..37381) 97%	2 (39445..39668) 94%	9 (41188..41521) 100%	0	0	1 41521	3 (42631..43095) 96%	8 (4..611) 100%	4 (608..1111) 94%	8 (1000..3288) *	3 (3288..4217) 97%	3 (4217..4802) 98%	3 (4802..5743) 97%	4 (5743..6414) 96%	8 (6414..6755) 98%	0	3 (6755..9249) 99%	3 (9249..9549) 99%	3 (9549..14241) 97%	0	0	2 (14241..14877) 99%	2 (14885..15084) 91%	1 (15084..19074) 92%	1 (19074..19627) 86%	2 (19627..20877) 99%	0	1 (20877..22535) 95%	1 (22696..23229) 99%	0	2 (23229..25092) 91%
F29	11	NC_004615	5 (83..1043) 99%	1 (1138..1185) 100%	4 (2901..3301) 99%	1 9380	8 (9918..10423) 94%	7 (10423..12204) 98%	0	0	2 (13703..13926) 97%	1 (14413..14702) 97%	6 (14702..14807) 99%	1 (14807..14923) 97%	1 14923	1 (15813..16370) 97%	1 (16377..16535) 98%	1 (16535..18241) 99%	1 (18241..21081) 97%	1 (21081..21358) 98%	1 (21360..25847) 97%	0	0	0	0	0	0	1 (25847..31543) 97%	0	0	1 (31543..32177) 93%	1 (32179..32400) 91%	1 (32403..36390) 92%	1 (36390..36942) 98%	1 (36942..38216) 95%	0	1 (38216..39874) 97%	1 (39969..40502) 96%	0	2 (40502..42365) 96%
F30	29	NC_007061	5 (27477..28437) 98%	1 (28532..28582) 98%	8 (30779..31225) 99%	1 35820	8 (36358..36863) 94%	7 (36863..38644) 97%	0	0	2 (39879..40107) 94%	1 (40358..40647) 97%	6 (40647..40752) 100	1 (40752..40868) 98%	1 40871	1 (42006..42564) 92%	2 (42585..480) 88-98	3 (480..1734) 97%	3 (1734..3168) 97%	3 (3168..4097) 96%	3 (4097..4682) 92%	8 (4682..5677) 99%	4 (5677..6348) 91%	8 (6348..6689) 96%	0	3 (6689..9188) 98%	8 (9188..9657) 99%	3 (9657..14349) 97%	0	0	2 (14349..14985) 98%	2 (15192..15192) 99%	1 (15192..19188) 90%	1 (19188..19741) 91%	2 (19741..20991) 98%	0	1 (20991..22649) 94%	1 (22744..23277) 97%	0	2 (23277..25140) 96%
F31	tp310-2	NC_009762	6 (103..1290) 96%	2 (1309..1389) 96%	4 (2514..2914) 99%	2 7226	0	2 (8226..11597) 99%	0	0	2 (12161..12390) 97%	1 (13870..14160) 96%	7 (14160..14281) 100%	0	1 14398	5 (18073..20032) 97%	5 (20032..21327) 92%	5 (21339..23564) 95%	5 (23564..29872) 99%	0	0	0	0	0	0	0	0	5 (29872..42631) 99%	0	0	0	0	0	1 (42631..43177) 86%	0	0	0	0	0	3 (43196..44627) 92%
F33	ROSA	NC_007058	6 (26815..28002) 98%	1 (28027..28077) 100%	8 (30155..30600)*	1 34475	4 (34677..35667)*	4 (35667..36149)*	1 (36149..36443) 98%	1 (36452..37295) 98%	2 (38921..39144) 94%	1 (40604..40894) 95%	4 (40894..41018)*	1 (41018..41135) 98%	1 41135	1 (42446..43003) 92%	4 (75..494)*	4 (497..1000)*	4 (1000..3177) *	2 (3177..4103) 94%	4 (4103..4706) *	4 (4706..5648)*	4 (5648..6319)*	4 (6319..6870)*	4 (6870..7836)*	2 (7836..10113) 100%	8 (10113..10582) 98%	2 (10582..15280) 99%	0	0	1 99%	2 (15924..161230 91%	1 (16123..20113) 92%	1 (20113..20666) 86%	2 (20666..21916) 99%	0	1 (21916..23574) 94%	1 (23669..24202) 97%	0	3 (24202..25633) 92%
F34	77	NC_005356	6 (21871..23058) 97%	1 (23083..23133) 98%	4 (24441..24841) 99%	1 31607	4 (31809..32799) 99%	8 (32799..33256) 100	1 (33256..33547) 93%	1 (33556..34399) 98%	2 (36027..36254) 93%	1 (37756..38046) 98%	5 (38046..38182) 100	1 (38182..38299) 98%	1 38299	6 (40499..40725) 99%	7 (40709..41705) 99%	7 (1..2120) 99%	7 (2120..5995) 98%	0	0	0	0	0	0	0	0	7 (5995..10444) 96%	2 (10444..11777) 95%	2 (11777..12574) 95%	4 (12574..13363) 100	4 (13363..14251) 99%	3 (14251..16851) 99%	0	4 (16851..17045) 98%	7 (17045..18723) 98%	2 (18723..18844) 99%	3 (18844..19237) 93%	0	3 (19258..20689) 93%
F35	NM4	DQ530362	6 (94..1281) 98%	1 (1306..1356) 100%	3 (3111..3324) 99%	1 8392	4 (8594..9584) 100%	4 (9584..10066) 100%	1 (10066..10360) 99%	1 (10369..11212) 99%	2 (12838..13061) 94%	3 (14615..14868) 95%	3 (14870..15161) 94%	0	1 15161	3 (16581..17045) 99%	2 (17056..17753) 99%	2 (17753..19038) 99%	2 (19038..20445) 99%	2 (20445..21373) 97%	2 (21373..23734)	0	0	4 (23734..24285) 99%	0	2 (24282..26558) 100	8 (26558..27027) 98%	2 (27027..31725) 99%	0	0	2 (31725..32361) 99%	2 (32369..32568) 91%	1 (32568..36558) 92%	1 (36558..37110) 98%	1 (37110..38384) 95%	0	1 (38384..40042) 97%	1 (40137..40670) 97%	0	3 (40670..42101) 92%
F39	53	NC_007049	7 (27644..29029) 99%	1 (29034..29084) 100%	4 (30606..31006) 99%	1 35557	4 (35759..36749) 100	4 (36749..37231) 100	1 (37231..37525) 99%	1 (37534..38377) 99%	2 (40003..40226) 94%	1 (41520..41809) 98%	8 (41809..42033)*	0	1 42033	1 (43326..43883) 99%	1 (7..165) 99%	1 (165..1871) 99%	1 (1871..4711) 95%	1 (4711..4988) 97%	1 (4990..9477) 98%	0	0	0	0	0	0	1 (9477..15170) 96%	0	0	1 (15170..15806) 96%	1 (15806..16027) 100	1 (16027..20023) 90%	1 (20023..20575) 92%	1 (20575..21849) 96%	0	1 (21849..23507) 96%	1 (23602..24135) 97%	0	3 (24135..25566) 92%
F40	85	NC_007050	7 (28601..29986) 100	1 (29991..30041) 98%	6 (31499..32086) 95%	1 37066	4 (37268..38258) 100	8 (38258..38715)*	1 (38715..39006) 92%	1 (39015..39858) 98%	2 (41484..41707) 94%	1 (43001..43291) 98%	5 (43291..43427) 100	1 (43427..43544) 98%	1 43544	1 (43721..44279) 98%	1 (3..160) 98%	8 (160..1955)*	1 (1955..4799) 95%	8 (4802..5096) 99%	1 (5096..9584) 98%	0	0	0	0	0	0	1 (9584..15277) 97%	0	0	1 (15277..15913) 99%	1 (15913..16134) 100	1 (16134..20130) 94%	1 (20130..20683) 94%	1 (20683..21957) 97%	0	1 (21957..23615) 98%	1 (23776..24309) 99%	0	2 (24309..25331) 90%
F41	NM2	DQ530360	7 (23..1408) 100%	1 (1413..1461) 98%	4 (2644..3044) 99%	1 8484	4 (8686..9676) 100%	4 (9676..10158) 100%	1 (10158..10452) 99%	1 (10461..11304) 99%	2 (12930..13153) 94%	3 (14707..14960) 95%	3 (14962..15253) 94%	0	1 15253	1 (15901..16458) 92%	1 (16465..16623)	1 (16623..18329) 99%	1 (18329..21170) 96%	8 (21173..21467)*	1 (21467..25956) 98%	0	0	0	0	0	0	1 (25956..31649) 98%	0	0	1 (31649..32283) 93%	1 (32285..32506) 90%	1 (32509..36496) 90%	1 (36496..37049) 96%	1 (37049..38323) 96%	0	1 (38323..39981) 98%	1 (40076..40609) 96%	0	2 (40609..42472)*
F42	92	NC_007064	7 (26248..27633) 99%	1 (27638..27688) 100%	4 (28556..28596) 99%	1 33758	4 (33960..34950) 100	4 (34950..35432) 99%	1 (35432..35726) 97%	1 (35735..36578) 97%	2 (38642..38865) 94%	9 (40385..40718)*	0	0	1 40718	3 (41827..42292) 96%	8 (1..608)*	4 (605..1108) 94%	8 (997..3285) 100%	3 (3285..4214) 97%	3 (4214..4799) 98%	3 (4799..5740) 97%	4 (5740..6411) 96%	8 (6411..6752) 98%	0	3 (6752..9246) 99%	3 (9246..9546) 99%	3 (9546..14238) 97%	0	0	2 (14238..14874) 99%	2 (14882..15081) 91%	1 (15081..19071) 92%	1 (19071..19624) 86%	2 (19624..20874) 99%	0	1 (20874..22532) 94%	1 (22627..23160) 95 %	0	2 (26268..27106) 92%
F43	P954	NC_013195	7 (25..1410) *	1 (1415..1465) 100%	7 (3387..3787) *	1 8953	0	7 (9945..11726)*	0	0	2 (13525..13748) 95%	1 (15041..15331) 97%	4 (15331..15455) 99%	1 (15455..15572) 99%	1 15572	7 (17722..17718)*	7 (17718..18714)*	7 (18714..20834)*	7 (20834..24686)*	0	0	0	0	0	0	0	0	7 (24686..29133)*	2 (29133..30466) *	2 (30466..31263)*	4 (31263..32052)*	4 (32052..32940)*	3 (32940..35540)*	0	4 (35540..35734)*	7 (35734..37412)*	2 (37412..37533)*	3 (37533..37923)*	2 (37953..38349)*	4 (38349..39363) 95%
